# Genetic Diversity and Population Genetic Analysis of *Plasmodium falciparum* Thrombospondin Related Anonymous Protein (TRAP) in Clinical Samples from Saudi Arabia

**DOI:** 10.3390/genes13071149

**Published:** 2022-06-25

**Authors:** Saad M. Bin Dajem, Md Atique Ahmed, Fatimah F. Alghnnam, Shouq F. Alghannam, Gauspasha Yusuf Deshmukh, Rehan Haider Zaidi, Marie Fe F. Bohol, Syeda Sabiha Salam, Syeda Wasfeea Wazid, Mohammed I. Shafeai, Fuad H. Rudiny, Ali M. Motaen, Kareem Morsy, Ahmed A. Al-Qahtani

**Affiliations:** 1 Department of Biology, College of Science, King Khalid University, Abha 61413, Saudi Arabia; saad1426@gmail.com (S.M.B.D.); kasayed@kku.edu.sa (K.M.); 2 ICMR-Regional Medical Research Center, Dibrugarh 786010, Assam, India; atiqbiotech@gmail.com; 3 Department of Infection and Immunity, Research Centre, King Faisal Specialist Hospital Research Centre, Riyadh 11211, Saudi Arabia; f.f.alghnnam@gmail.com (F.F.A.); shouq.alghnnam@gmail.com (S.F.A.); mbohol@kfshrc.edu.sa (M.F.F.B.); 4 Department of Biotechnology and Microbiology, National College, Tiruchirapalli 620001, Tamil Nadu, India; gauspashabt17@nct.ac.in (G.Y.D.); rehanbt15@nct.ac.in (R.H.Z.); 5 Department of Life Sciences, Dibrugarh University, Dibrugarh 786004, Assam, India; syedasabihasalam1987@gmail.com; 6 Arogya Society of Health, Welfare and Support (ASHWAS), Dinsugia 785640, Assam, India; wasfeea@gmail.com; 7 Sabya General Hospital, Sabya 85534, Saudi Arabia; mshafeai@moh.gov.sa (M.I.S.); frudiny@moh.gov.sa (F.H.R.); almotaen@gmail.com (A.M.M.); 8 Department of Microbiology and Immunology, College of Medicine, Alfaisal University, Riyadh 11533, Saudi Arabia

**Keywords:** genetic diversity, *Plasmodium falciparum*, natural selection, population structure, genetic differentiation

## Abstract

The thrombospondin related anonymous protein (TRAP) is considered one of the most important pre-erythrocytic vaccine targets. Earlier population genetic studies revealed the *TRAP* gene to be under strong balancing natural selection. This study is the first attempt to analyze genetic diversity, natural selection, phylogeography and population structure in 199 clinical samples from Saudi Arabia using the full-length *PfTRAP* gene. We found the rate of nonsynonymous substitutions to be significantly higher than that of synonymous substitutions in the clinical samples, indicating a strong positive or diversifying selection for the full-length gene and the Von Willebrand factor (VWF). The nucleotide diversity was found to be π~0.00789 for the full-length gene; however, higher nucleotide diversity was observed for the VWF compared to the thrombospondin repeat region (TSP). Deduction of the amino acid sequence alignment of the PNP repeat region in the Saudi samples revealed six genotypes characterized by tripeptide repeat motifs (PNP, ANP, ENP and SNP). Haplotype network, population structure and population differentiation analyses indicated four distinct sub-populations in spite of the low geographical distance between the sampling sites. Our results suggest the likeliness of independent parasite evolution, creating opportunities for further adaptation, including host transition, and making malaria control even more challenging.

## 1. Introduction

Malaria persists as a serious public health issue, with *P. falciparum* being the most virulent cause accounting for the majority of cases globally. According to global malaria statistics, there are an estimated 228 million malaria cases and 405,000 deaths worldwide each year [1,2]. Although there was a gradual decline in malaria cases in 2017, malaria still remains a major public health concern [3]. As per the World Malaria Report 2021, a sudden rise in malaria death cases was observed between 2019 and 2020 due to disruptions in malaria control because of the COVID-19 pandemic [4]. The issue was further compounded due to the parasite’s widespread resistance to commonly used antimalarial drugs, as well as the absence of a licensed vaccine, which together contribute to the present impact of the disease [5,6]. All these factors impose on malaria researchers the core responsibilities of continuing to develop antimalarial drugs and identifying antigens as possible malaria vaccine candidates.

In Saudi Arabia, over the past twenty years, the malaria control program has efficiently lowered the incidence of malaria annually. However, disease infections are reported to still exist, and they have been increasing since 2016 [7] in the regions located along the Red Sea, particularly in the Jazan province, where *Anopheles arabiensis* and *A. sergentii* continue to cause transmission [8,9,10]. Among the seven countries in the Arabian Peninsula, Saudi Arabia and Yemen remain the only two countries that are yet to achieve malaria elimination [8,9,11]. Of the five species of *Plasmodium* that are known to transmit to humans, *P. falciparum* accounts for the majority of the total malaria cases in Saudi Arabia (i.e., 99%), and only 1% are attributable to *P. vivax* [12]. However, the malaria incidence from *P. vivax* has been reported to be rising since 2015, as seen from data from India, Pakistan and Ethiopia [13]. According to World Health Organization (WHO) statistics, during the period from 2010 to 2015, the total number of malaria cases in Saudi Arabia was recorded to be lower than 100, and it increased to 272 in 2016 [14]. Previous reports reveal a higher percentage of disease infections among non-Saudi patients, with the majority of the cases being among Yemeni patients, followed by Pakistanis, Nigerians and Indians [8,10,15,16]. Urbanization, as well as urban settlement patterns and the need for urban extension, might be reasons substantially influencing malaria transmission in Saudi Arabia. In addition, poor public awareness, along with parasite and vector resistance to treatments and insecticides, respectively, are also reported to be factors leading to the prevalence of the disease [7,8,17].

Malaria infection in humans begins when female *Anopheles* mosquitoes bite humans and inject sporozoites. Sporozoites rapidly move to the liver and start infecting hepatocytes [3]. As malaria infection in humans is initiated by sporozoites, sporozoites are viewed as key targets for pre-erythrocytic malaria vaccines [18,19,20]. The principal strategy to control and eliminate malaria is the development of an effective antimalarial vaccine. However, antigenic variation is a major obstacle when designing vaccines for *Plasmodium* spp. and non-malarial pathogens, including the HIV virus, hepatitis C virus, Ebola virus, dengue virus, etc., thus widening the scope of the challenge [21]. The leading malaria vaccine antigens, such as AMA1, MSP1 and MSP2, are mainly parasite surface proteins and have been found to develop substantial genetic diversity to escape host immune responses [22,23]. The effectiveness of these vaccine candidates was found to be diminished when they were examined in field trials, due to the allele-specific immune response displayed [24]. In malaria-endemic regions, the extent of the nucleotide diversity is directly proportional to the degree of transmissibility [25].

During the parasite hepatic phase, various surface proteins, such as *P. falciparum* circumsporozoite protein (CSP), thrombospondin related anonymous protein (TRAP) and liver-stage antigens (LSAs), are released from the sporozoite surfaces as soon as they are exposed to the host cell [26]. However, CSP and TRAP are considered to be the most important vaccine targets located on the sporozoite surfaces [27,28]. The attenuated sporozoite vaccine PfSPZ is being trialed in the immunization of human volunteers against falciparum malaria [29]. Although several vaccine trials are underway, recent trial reports indicate poor efficacy, except for the malaria subunit vaccine RTS,S (Mosquirix) targeting CSP protein, which produced immunity against sporozoites [30]. Unfortunately, RTS,S produced low clinical efficacy in *P. falciparum* strains, which was ascribed to the vast parasite diversity and allele-specific immune response detected among field isolates [31]. However, recently, the WHO stated that the vaccine could prevent 40% of malaria cases and 30% of severe malaria cases in young African children [32]. TRAP is a type-I transmembrane protein located in the micronemes of sporozoites [33,34]. It plays a crucial role in sporozoite gliding motility and invasion of liver cells [26,33,35]. A previous report reveals that interference in the *PfTRAP* gene by gene knockout interrupts sporozoite gliding movement and invasion of the salivary gland [36]. Due to its importance in *Plasmodium* proliferation, this gene is the most widely studied *Plasmodium* transmembrane protein. The extracellular domain of *PfTRAP* is comprised of three motifs: the A-motif, the thrombospondin repeat (TSR) motif and a proline-rich portion at the C-terminal [3]. Von Willebrand factor A (VWA) is another motif that is also present in the ectodomain of *PfTRAP*. Part of the VWA motifs contain the inserted domain (I) as integrins, constituting metal ion-dependent adhesion sites (MIDAS) attached with a Mg^2+^ ion at the central position of the ligand-binding site [18,37]. The TRAP VWA motif comprises the sequence signature of a MIDAS. Earlier reports have revealed mutations in putative TRAP VWA motif MIDAS residues, and deletion of a part of the TRAP TSR motif interrupts parasite gliding movement and invasion of invertebrate salivary glands and vertebrate hepatic cells [36,38].

A previous study on the Gambian and Thai populations reported high frequencies of nonsynonymous to synonymous single-nucleotide polymorphisms (SNPs) of TRAP [35]. This study’s reports on Tajima’s and Fu and Li’s tests indicated the *TRAP* gene to be acting under diversified selection on the genes of both *PfTRAP* and *PvTRAP*. Earlier studies revealed strong diversifying selection for the *TRAP* gene in both *P. falciparum* and *P. vivax*, indicating *TRAP* to be an attractive vaccine target [35,39,40]. However, no prior studies have been undertaken to understand the genetic differentiation of the *PfTRAP* gene in clinical samples from Saudi Arabia. Therefore, the present study aimed to elucidate the genetic diversity, natural selection and population structure of *PfTRAP* from 199 clinical samples from Saudi Arabia, along with sequences from three other countries (i.e., Malaysia, Thailand and Gambia), using the full-length *PfTRAP* gene.

## 2. Materials and Methods

### 2.1. Sample Collection

**Figure 1:**
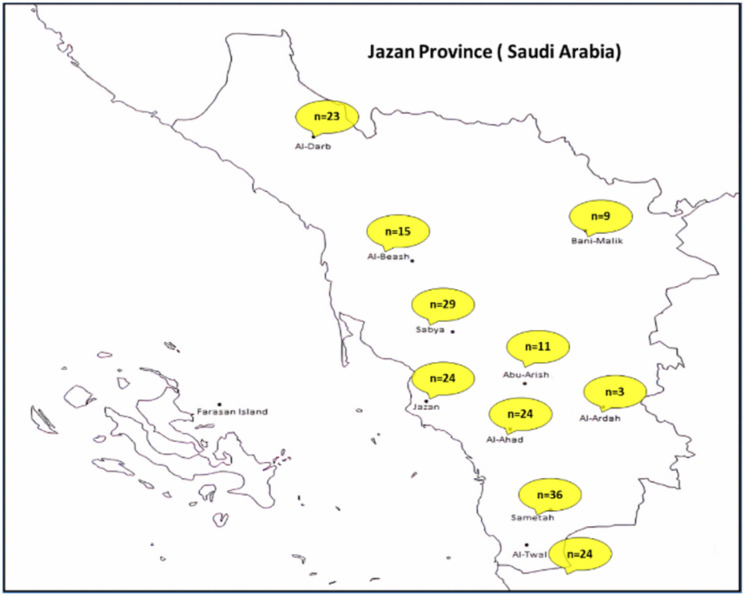
Sample collection sites and sequences generated from each sites.

The present study was approved by the Ethical Review Committee of Research of the Ethical Review Board (ERB) of King Fahad Central Hospital (KFCH), Jazan. For the study, finger-pricked blood samples were collected on filter paper from 440 RDT-positive patients infected by *P. falciparum*. These were patients attending various health clinics in the Jazan province (*viz.* Al-Darb, Bani-Malik, Beash, Sabya, Abu-Arish, Jazan, Al-Ahad, Al-Ardah, Sametah and Al-Twal) (Figure 1). Informed consent was obtained from all the patients prior to blood sampling. For microscopic detection, both thin and thick blood smears were collected using Giemsa staining. Information on parasite counts, age and sex obtained in the study is provided in Supplementary Table S1.

### 2.2. DNA Extraction and PCR Amplification

DNA extraction was carried out from whole blood using a DNeasy Blood and Tissue Kit (Qiagen GmbH, Hilden, Germany) according to the manufacturer’s instructions.

#### Polymerase Chain Reaction (PCR), Amplification and DNA Sequencing

The *TRAP* gene was amplified in three separate segments. Nucleotide sequences for all primers used in the amplification of the three segments are shown in Table 1. Due to difficulties in amplifying segments 1 and 3, nested PCR procedures were used for successful amplification of these two segments. M13 primers were added to the amplifying primers to facilitate sequencing.

The following PCR amplification conditions were used for segment 1. For round one, the PCR mixture included 5 µL of DNA template, 1X final concentration of GoTaq Green Master Mix (Promega, Madison, WI, USA) and 0.2 µM for each primer (S1_For-1 and S1_Rev-1), with 30 µL final volume. For the nested amplification, 2 µL of round one amplicon was used, along with 1X final concentration of GoTaq Green Master Mix (Promega) and 0.16 µM for each primer (S1_For-2 and S1_Rev-2), with 30 µL final volume. Cycling parameters for both rounds 1 and 2 were 95 °C for 5 min, followed by 40 cycles of 95 °C for 30 s, 56 °C for 30 s and 72 °C for 90 s and a final extension at 72 °C for 5 min. For segment 2, 2 µL of DNA template was used, along with 1X final concentration of GoTaq Green Master Mix (Promega) and 0.125 µM for each primer, with 40 µL final volume. Cycling parameters were 95 °C for 5 min, followed by 45 cycles of 95 °C for 30 s, 56 °C for 30 s and 72 °C for 30, and a final extension at 72 °C for 5 min. For segment 3, the mixture for round one included 5 µL of DNA template, 1X final concentration of GoTaq Green Master Mix (Promega) and 0.13 µM for each primer (S3_For-1and S3_Rev-1), with 30 µL final volume. For the nested amplification, 1.5 µL of round one amplicon was used, along with 1X final concentration of GoTaq Green Master Mix (Promega) and 0.1 µM for each primer (S3_For-2 and S3_Rev-2), with 30 µL final volume. Cycling parameters were 95 °C for 5 min, followed by 40 cycles of 95 °C for 30 s, 57 °C for 20 s and 72 °C for 30 s and a final extension at 72 °C for 5 min. PCR products were run and visualized on 2% agarose gel.

PCR products were gel-purified and subjected to dideoxy Sanger sequencing using forward and reverse M13 as sequencing primers. The resulting sequencing chromatograms were assembled using SeqMan Pro 15 (DNASTAR Lasergene, DNAStar Inc., Madison, WI, USA) and a consensus FASTA file was saved in EditSeq 15 (DNASTAR Lasergene). MegAlign 15 (DNASTAR Lasergene) was used to perform multiple sequence alignment of nucleic acids and/or proteins.

### 2.3. Sequence Diversity and Natural Selection

In this study, sequence diversity (π) was determined using DnaSP v5.10 software [41]. The numbers of polymorphic sites, parsimony informative sites, synonymous (silent mutations) and nonsynonymous substitutions (replacement changes), haplotypes (H) and singletons and the nucleotide and haplotype diversity for the full-length *PfTRAP* gene and the reference strain PF3D7_1335900were determined using DnaSP v5.0 software [41]. Domain-wise analyses for the TSP region and Von Willebrand factor were also evaluated using DnaSP software. Analyses were conducted using samples from Saudi Arabia (n = 199) and three other countries: Malaysia (n = 3), Thailand (n = 32) and Gambia (n = 7). Alignment of the sequences was undertaken using the CLUSTAL-W program in MegAlign, Lasergene v 7.0 (DNASTAR), while polymorphism and phylogenetic analyses were performed with MEGA 5.0 software [42]. Using a window length of 100 and a step size of 25 bp in DnaSP, nucleotide diversity was also graphically determined. Further, to calculate the rate of nonsynonymous substitution per nonsynonymous site (dS) and the rate of synonymous substitution per synonymous site (dN), Nei and Gojobori’s method, which determines natural selection, was used in MEGA 5.0. Additional tests for natural selection, Tajima’s D and Fu and Li’s D* and F* neutrality tests, were performed using DnaSP v5.10. Tajima’s D value is zero for neutral evolution, a negative value of Tajima’s D corresponds to negative selection or population expansion and a positive value or significant value corresponds to positive or balancing selection. The Tajima’s D values were also graphically illustrated using the DnaSP software. Positive values for Fu and Li’s D* and F* denote population contraction, whereas negative values denote population expansion and singleton excess.

### 2.4. Haplotype Network and Population Structure Analysis

For the haplotype network and population structure analyses, we categorized the sampling locations from Saudi Arabia into five regions based on distances of 50 km (i.e., region 1 was Bani-Malik; region 2 was Beash; region 3 was Al-Ahad, Sametah and Al-Twal; region 4 was Abu-Arish, Sabya, Jazan and Al-Ardah; and, lastly, region 5 was Al-Darb). For haplotype network analysis, 42 samples from Malaysia, Thailand and Gambia were also included. The haplotype network for the *PfTRAP* gene was created using the median-joining method in NETWORK version 4.6.1.2 software (Fluxus Technology Ltd., Suffolk, UK). The genetic structure of the *Plasmodium falciparum* parasite population was determined using STRUCTURE V2.3.4 software, which is based on a Bayesian Markov chain Monte Carlo (MCMC) model [43]. Using an admixture model (K), the most likely number of populations was computed. K was set from 1 to 10, and a single run was conducted. Following a burn-in period of 50,000 steps, we used 500,000 Markov chain Monte Carlo generations for each run. From the data, the most probable K-value was predicted by evaluating ΔK values based on the rate of change in log probability (LnP(D)) between successive K-values, using the STRUCTURE Harvester [43]. Re-analyses of the population structure of *PfTRAP* were also conducted by removing the P-N-P regions from the genes using STRUCTURE software. The pairwise differences (*F_ST_*) between populations were also computed using ARLEQUIN version 3.5.1.3 [44]. *F_ST_* compares the frequencies of alleles within and between populations to calculate genetic variability within and between populations. *F_ST_* can be interpreted as no genetic differentiation when values are 0, low differentiation at <0–0.05, moderate at 0.05–0.15 and high at 0.15–0.25.

## 3. Results

### 3.1. Schematic Structure and Nucleotide Diversity within PfTRAP Sequences

**Figure 2:**
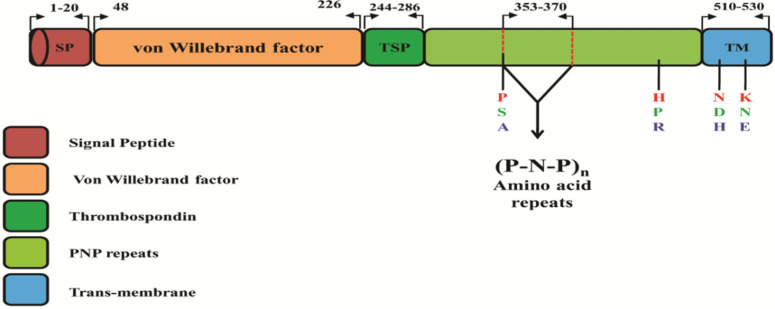
Schematic representation of the *P. falciparum* full-length *PfTRAP* gene in the 3D7 strain (PF3D7_1335900; 1725bp), indicating the different domains of the gene and the position of the tri-variants. The numbers on the top with the bended arrows represent positions of the characterized domains.

**Figure 3:**
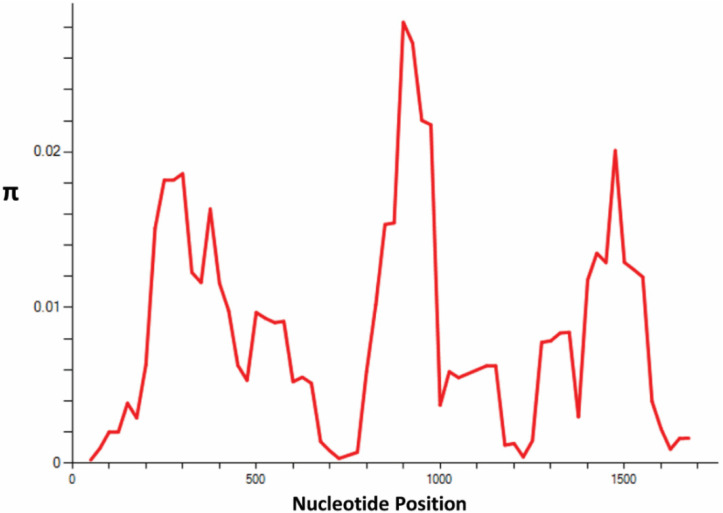
Illustration of nucleotide diversity (π) in the region between 900 and 1000 nt for the *PfTRAP* gene. The graph was generated using DnaSP software version 5.0, with a window length and step size of 100 and 25, respectively.

The schematic structure of *PfTRAP*, a single exon that contains specialized domains, such as the von Willebrand factor, thrombospondin type (TSP) repeat and (P-N-P)n repeats, is shown in Figure 2. Of the 440 clinical samples, high quality full-length TRAP gene sequences were obtained from only 199 samples. Samples that did not yield full-length high quality sequences were not included for downstream analysis. The sequences generated from this study were submitted to NCBI with the following accession numbers: ON332256–ON332454. Analysis of the nucleotide alignment of 199 sequences from Saudi Arabia, along with the 3D7 reference strain, revealed that there were 175 single nucleotide polymorphisms (SNPs), 36 of which were synonymous and 130 nonsynonymous substitutions Table 2. The overall nucleotide diversity for the full-length *PfTRAP* gene was found to be π = 0.00789 ± 0.00027, with seven singleton variable sites that were tri-variants. The nucleotide diversity of the TSP domain was lower than that of the Von Willebrand factor (VWF) at 0.00580 ± 0.00048 and 0.00971 ± 0.00033, respectively (Table 2). A graphical representation of the nucleotide diversity is shown in Figure 3. Sequence analysis of the *PfTRAP* gene revealed 121 singletons, 62 parsimony informative sites and 103 haplotypes, with a haplotype diversity of 0.096 ± 0.005. Within the gene, there were four hypervariable amino acid polymorphisms observed: P353S/A, H469P/R, N508D/H and K511N/E (Figure 2). The amino acid polymorphism within Saudi Arabian sequences is shown in Supplementary Figure S1. The nucleotide alignment of 42 full-length sequences from three countries (Malaysia, Thailand and Gambia), along with the 3D7 reference strain, of the *PfTRAP* gene showed 65 single nucleotide polymorphisms (SNPs), of which 2 were synonymous and 63 nonsynonymous substitutions (Table 2). The overall nucleotide diversity (π) was found to be 0.00768 ± 0.00059 and the number of haplotypes was found to be 33, with a haplotype diversity of 0.975 ± 0.015.

The overall nucleotide diversity of the 241 samples comprising samples from Saudi Arabia, Malaysia, Thailand and Gambia revealed 190 SNPs, of which 37 were synonymous and 153 nonsynonymous substitutions (Table 2). The overall nucleotide diversity was found to be 0.0084 ± 0.00023, and that for the TSP domain was found to be lower than that for the Von Willebrand Factor as observed in the Saudi Arabian sequences (Table 2).

### 3.2. Natural Selection and Genetic Diversity in PfTRAP Gene

**Figure 4:**
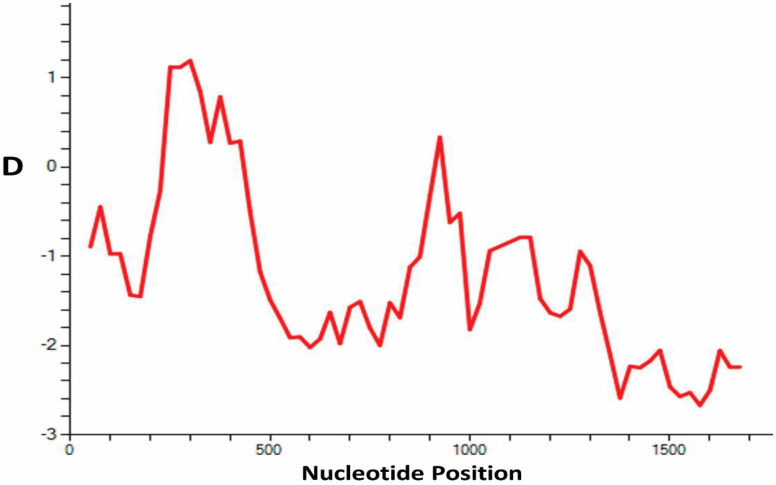
Illustration of Tajima’s D values (D) for the full-length *PfTRAP* gene among the clinical samples from Saudi Arabia. The graph was generated using DnaSP software version 5.0, with a window length and step size of 100 and 25, respectively.

The natural selection analysis of the full-length *PfTRAP* gene for the 199 clinical samples using the codon-based z-test for positive selection revealed that the gene is under strong positive or diversifying selection (dN-dS = 5.386, *p* < 0.00). Domain-wise analysis also showed significant positive natural selection (dN-dS = 4.018, *p* < 0.00) for the Von Willebrand Factor; however, the TSP domain was not significant (Table 1). The overall Tajima’s D value was found to be negative and significant, which indicates population expansion (D = −1.918, *p* < 0.05) (Table 2). A graphical representation of the Tajima’s D value is depicted in Figure 4. Similarly, Fu and Li’s D* (−8.788, *p* < 0.05) and F* (−6.526, *p* < 0.05) values were found to be negative and significant (Table 2). Analysis of natural selection among samples from Malaysia, Thailand and Gambia also revealed that the gene has been exposed to positive or diversifying selection (dN-dS = 6.29, *p* < 0.05). Natural selection analysis of 241 *PfTRAP* sequences revealed similar results, with VWF showing strong natural selection (Table 1).

### 3.3. Proline-Asparagine-Proline (P-N-P) Repeat Characterization

**Figure 5:**
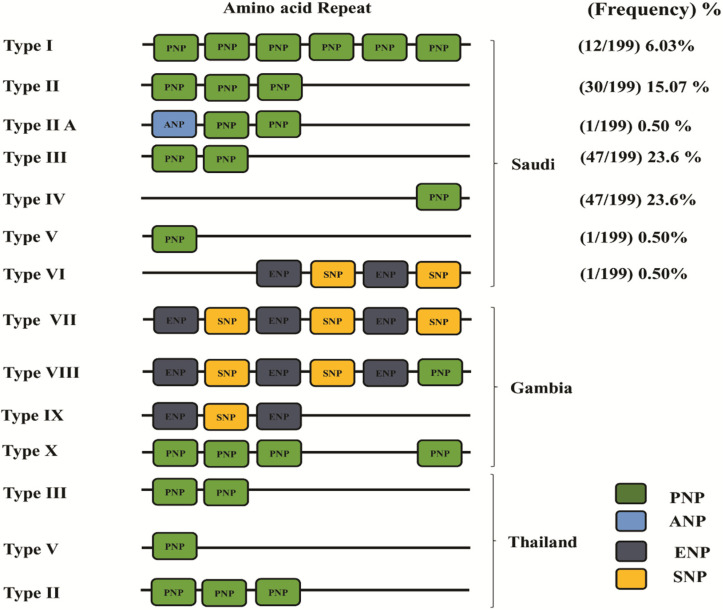
The PNP repeat types observed in the 199 PfTRAP amino acid sequences from Saudi Arabia and their frequencies. Each tripeptide repeat motif is colored using four different colors. Samples with complete deletion of the PNP region (n = 60) are not shown.

Deduced amino acid sequence alignments were used to characterize the tripeptide repeat unit in the *PfTRAP* clinical samples from Saudi Arabia, Malaysia, Thailand and Gambia. In this study, in addition to the PNP motifs, we identified three other novel tripeptide repeat motifs (ANP, ENP and SNP). In accordance with the arrangement of PNP repeat motifs and the three novel tripeptide motifs, ten genotypes were identified (named Type I to Type X in this study). Among the Saudi samples, six genotypes (Type I to Type VI) were observed, with highest prevalence for the Type III (23.6%) and Type IV (23.6%) genotypes, followed by Type II (15.07%), Type I (6.03%) and, lastly, Type IIA, Type V and Type VI, which had similar and equal prevalence (i.e., 0.50%), respectively (Figure 5). Type II, Type III and Type V genotypes were also found in Thailand samples, whereas no similarity was found for the Gambian samples (Figure 4).

### 3.4. Haplotype Network and Population Structure Analysis

**Figure 6:**
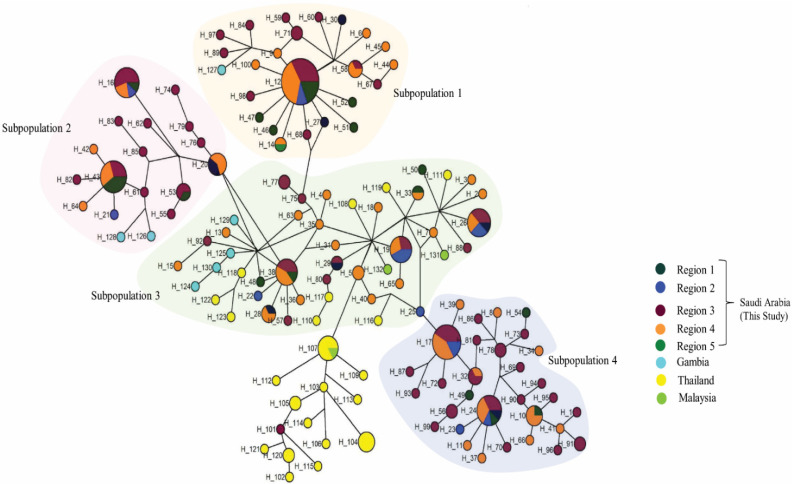
Haplotype network analysis of 132 *PfTRAP* from Saudi Arabia (regions 1 to 5), Thailand and Malaysia. The shaded clusters represent the four different sub-populations observed within the haplotypes. Circles represent haplotypes with sizes proportional to haplotype frequency.

**Figure 7:**
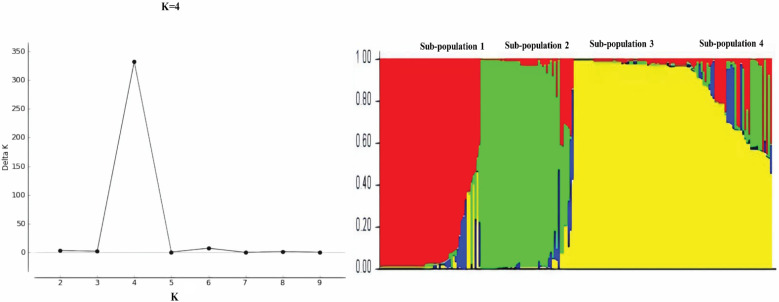
The most likely numbers of *PfTRAP* parasite sub-populations within the 199 clinical samples from Saudi Arabia. The numbers of populations were estimated using the delta K value (ΔK), and four distinct sub-populations were observed (K = 4, ΔK = 332.03). The line graph of the population structure is shown using membership coefficients (Q values).

The haplotype network analysis of 132 haplotypes of the *PfTRAP* gene revealed no geographical clustering. However, genetic clusters were observed, which grouped samples from Saudi Arabia into four sub-populations (Figure 6). A major shared haplotype cluster (Hap_12, n = 22, sub-population 1) was observed, comprised of the samples from four sampling regions (regions 1 to 4) (Figure 6). Minor shared haplotype clusters (H_17, H_43, H_16, H_38, H_24, H_26, H_107 and H_19) were observed in the remaining three sub-populations. The samples from Thailand and Malaysia shared genetic clustering with sub-population 3 (Figure 6). A Bayesian population structure analysis of the 199 *PfTRAP* clinical samples from Saudi Arabia revealed that there were four distinct sub-populations in Saudi Arabia (K = 4, ΔK = 332.03) (Figure 7). Re-analysis of *PfTRAP* sequences, removing the highly variable repeat region (P-N-P)n, also resulted in four sub-populations (K = 4) (Supplementary Figure S2).

The population differentiation analysis fixation index (*F_ST_*) obtained between region 2 (Beash) and region 3 (Al-Ahad, Sametah and Al-Twal) equaled 0.981 with *p* < 0.05, which was significant, and the lowest Fst value was observed between region 1 (Bani-Malik) and 5 (Al-Darb) (Fst = 0.022, *p* < 0.05) (Table 3). A moderate Fst was found for regions 2 and 5. However, most of the fixation indices among the sub-population showed high and significant genetic differentiation, even though they were separated by low geographical distance. This might have been due to multiple recombination events occurring in the chromosome as a result of multiple genetic clusters observed. These clustering patterns between the clinical samples were consistent with the level of genetic differentiation observed among the defined geographical regions. This showed that genetic differentiation among these samples was significant, resulting in four sub-populations despite the parasite being limited to Saudi Arabia. Additionally, the NETWORK analysis of these samples revealed that there were four distinct genetic clusters.

## 4. Discussion

Among the hepatic-stage antigens, which are localized on sporozoite surfaces, PfTRAP plays a significant role in sporozoite gliding movement and invasion into liver cells. During release of sporozoites into blood, these antigens are directly exposed to host immune responses and, therefore, are considered potential vaccine candidates and are being assessed in various clinical trials [3,45,46,47]. In the context of Saudi Arabia, no studies have been undertaken to genetically characterize the *PfTRAP*, gene which is one of the leading malaria vaccine candidates. Therefore, this study is the first attempt to understand genetic variations, natural selection and population structure in clinical samples obtained from the port region of Jazan, Saudi Arabia.

Moreover, the highly diverse nature of the parasite population revealed from this study is suggestive of a high degree of transmissibility and may pose a barrier for malaria control efforts in the region. The high antigenic diversity and the creation of new haplotypes correlated to transmission intensity, which might have been due to the presence of multi-clonal infections and multiple recombination events at the parasite sexual stage [25]. Moreover, four hyper-variable amino acid polymorphisms were observed within the *PfTRAP* exon. The results obtained from this study showed positive and significant selection for the full-length gene (dN-dS = 5.386) and VWF domain (dN-dS = 4.018). This was due to the significantly higher value in the nonsynonymous substitution rate as compared to the synonymous substitution rate. The (P-N-P)n repeat region of the *PfTRAP* gene showed different allele frequencies of varying PNP repeats. The PNP repeats of Saudi Arabia and Thailand exhibited similar repeat types, while those of Gambian clinical isolates exhibited variations in the P-N-P repeat region.

Noticeably, in spite of the low geographical distance between the sampling sites in Saudi Arabia, strong and significant genetic differentiation was observed, resulting in four sub-populations (K = 4). This may imply that the migrant population has a role in transmitting the infection, leading to local transmission. The haplotype network analysis revealed that the clinical samples had high genetic differentiation, which led to clustering of four different sub-populations. Analysis of population differentiation between parasite populations originating from different locations of Saudi Arabia showed high genetic differentiation. Despite having low nucleotide diversity (π = 0.0078) for the *PfTRAP* gene, significant and high Fst values were obtained between the different geographical locations of Saudi Arabia. Although regions 2 and 3 had low geographical separation, interestingly, the genetic differentiation between them was found to be high (Fst = 0.981; *p* < 0.05). Previous reports have revealed an increase in malaria transmission during the last few years in Saudi Arabia. The rise in the cases has been reported to be possibly related to the constant flow of immigrants from India, Pakistan, Sudan and Yemen [48]. Yemen is one of the poorest and most underdeveloped countries in the Arabian Peninsula, with the highest malaria incidence and transmission [14]. The high population migration from the war zones of Yemen and the difficulties in supplying sufficient medical services in those regions has also been reported to affect the importation of infections to neighboring countries. Currently, Yemen imposes a serious risk for imported malaria infections [10]. Furthermore, the likelihood of recombination between partially differentiated parasite genomes creates opportunities for further adaptation, including additional host transitions, making malaria control even more challenging.

## 5. Conclusions

This study is the first attempt to understand the genetic diversity and population genetic structure of the *PfTRAP* gene using clinical samples from Saudi Arabia. The levels of polymorphism within the sequences were moderate; however, a high level of haplotype diversity was noted, indicating the population expansion of the parasite. Multiple analyses found four distinct sub-populations within the Jazan province of Saudi Arabia, indicating that any TRAP-based vaccine has to consider the alleles defining these sub-populations. The data presented and discussed in this study will be helpful in understanding and rationally designing a TRAP-based vaccine for malaria.

## Figures and Tables

**Table 1:** Primers used in the amplification and sequencing of the TRAP gene.

Primer Name	Primer Sequence *
S1_For-1	AGG AAG AAC GTC TAA TAT ACA TA
S1_Rev-1	GGA AAT GAC GGT GAA GGA GA
S1_For-2	GTA AAA CGA CGG CCA GTA TAA TTT GTA TGT GCA TGC GTA C
S1_Rev-2	CAG GAA ACA GCT ATG ACC TTC AAC ACA AAC AGC CTT CA
S2_For	GTA AAA CGA CGG CCA GTA TCG TGG TGT TAA AAT AGC TGT
S2_Rev	CAG GAA ACA GCT ATG ACC GGG GTC ACT TTG TTT CCT TT
S3_For-1	AGA AGA AGG AAA GGG TGA AAA TC
S3_Rev-1	AAC ACA ATC TTA TTT CTC TTG CTA C
S3_For-2	GTA AAA CGA CGG CCA GTA AAC CCG AAA ATA AGC ACG AT
S3_Rev-2	CAG GAA ACA GCT ATG ACC AAC ACA ATC TTA TTT CTC TTG CT

* M13 sequences used for sequencing are underlined.

**Table 2:** Data for nucleotide diversity, haplotype diversity and the natural selection test for the *PfTRAP* gene and its domains.

Location	Domain	No. of Samples	SNPs	Syn	NonSyn	No. of Haplotypes	Diversity ± SD		Codon-Based *z*-Test (dN─dS)	Fu and Li’s D*	Fu and Li’s F*	Taj D
							Haplotype	Nucleotide				
Saudi Arabia (n = 199)	Full length	199	175	36	130	103	0.976 ± 0.005	0.00789 ± 0.00027	5.386 (*p* = 0.00)	−8.78807 (*p* < 0.02)	−6.52681 (*p* < 0.02)	−1.91853 (*p* < 0.05)
	TSP	199	14	3	11	9	0.597 ± 0.019	0.00580 ± 0.00048	1.251 (*p* = 0.107)	−547467 (*p* < 0.02)	−4.85951 (*p* < 0.02)	−1.70608 (*p* > 0.05)
	VWF	199	46	3	43	46	0.914 ± 0.010	0.00971 ± 0.00033	4.018 (*p* = 0.00)	−5.00266 (*p* < 0.02)	−3.98593 (*p* < 0.02)	−1.15329 (*p* > 0.10)
Malaysia, Thailand and Gambia (n = 42)	Full length	42	65	2	63	33	0.975 ± 0.015	0.00768 ± 0.00059	6.299 (*p* = 0.00)	−1.47503 (*p* > 0.10)	−1.44789 (*p* > 0.10)	−0.7500 (*p* > 0.10)
Overall (Saudi Arabia, Malaysia, Thailand and Gambia) (n = 241)	Full length	241	190	37	153	132	0.982 ± 0.003	0.00844 ± 0.00023	5.666 (*p* = 0.00)	−9.39721 (*p* < 0.02)	−6.75398 (*p* < 0.02)	−1.90977 (*p* < 0.05)
	TSP	241	17	3	14	12	0.597 ± 0.017	0.00570 ± 0.00042	1.291 (*p* = 0.10)	−5.92031 (*p* < 0.02)	−5.22152 (*p* < 0.02)	−1.87005 (*p* < 0.05)
	VWF	241	54	4	50	63	0.938 ± 0.007	0.01038 ± 0.00030	4.122 (*p* = 0.0)	−5.95795 (*p* < 0.02)	−4.59354 (*p* < 0.02)	−1.23611 (*p* > 0.10)

SD; standard deviation, Syn; synonymous substitutions, NonSyn; nonsynonymous substitutions.

**Table 3:** Population differentiation values (FST) for *PfTRAP* from Saudi Arabia.

Location	F_ST_ Values				
	Region 1	Region 2	Region 3	Region 4	Region 5
Region 1	-	-	-	-	-
Region 2	0.08255 ± 0.0029 *	-	-	-	-
Region 3	0.33155 ± 0.0251	0.98137 ± 0.0041 *	-	-	-
Region 4	0.37933 ± 0.0165	0.93230 ± 0.0064 *	0.65881 ± 0.0228	-	-
Region 5	0.02222 ± 0.0015 *	0.12407 ± 0.0073 *	0.69787 ± 0.0181	0.89582 ± 0.0154	-

* indicates significant values (*p* < 0.05).

## Data Availability

The DNA sequences generated in this study are available with NCBI accession numbers ON332256-ON332454.

## Supplementary Materials

The following supporting information can be downloaded at: https://www.mdpi.com/article/10.3390/genes13071149/s1, Figure S1: Amino acid polymorphism within PfTRAP sequences from Saudi Arabia; Figure S2: The numbers of populations were estimated using the delta K value (ΔK), and four distinct sub-populations were observed (K = 4, ΔK = 41.48); Table S1: Information on parasite counts, age and sex obtained in the study.
