# Supplementary material for: Genetic Diversity and Population Genetic Analysis of Plasmodium falciparum Thrombospondin Related Anonymous Protein (TRAP) in Clinical Samples from Saudi Arabia
Source: Genes (Basel). 2022 Jun 25;13(7):1149. doi: 10.3390/genes13071149 (PMC9319867; doi:10.3390/genes13071149)
Supplement: Supplementary file 1 [file genes-13-01149-s001.zip › genes-1726515-supplementary/Supplementary Table S1_revised.pdf]

Supplementary Table S1: Baseline characteristics of 440 *P. falciparum* infected patients enrolled in the study.

Variables	Value
Age	
Mean ± SD	28.01 ± 13.95
Median (25th-75th)	25.50 (19.0-36.75)
Sex	
Male count %	374 (85.03%)
Female count %	66 (14.97%)
Parasite density*	
Low	67 (15.22%)
Moderate	94 (21.32%)
High	279 (63.4%)

* WHO. Basic Malaria Microscopy. 2. Geneva: World Health Organization; 2010.
