# Supplementary figures and images for: Genetic Diversity and Population Genetic Analysis of Plasmodium falciparum Thrombospondin Related Anonymous Protein (TRAP) in Clinical Samples from Saudi Arabia
Source: Genes (Basel). 2022 Jun 25;13(7):1149. doi: 10.3390/genes13071149 (PMC9319867; doi:10.3390/genes13071149)

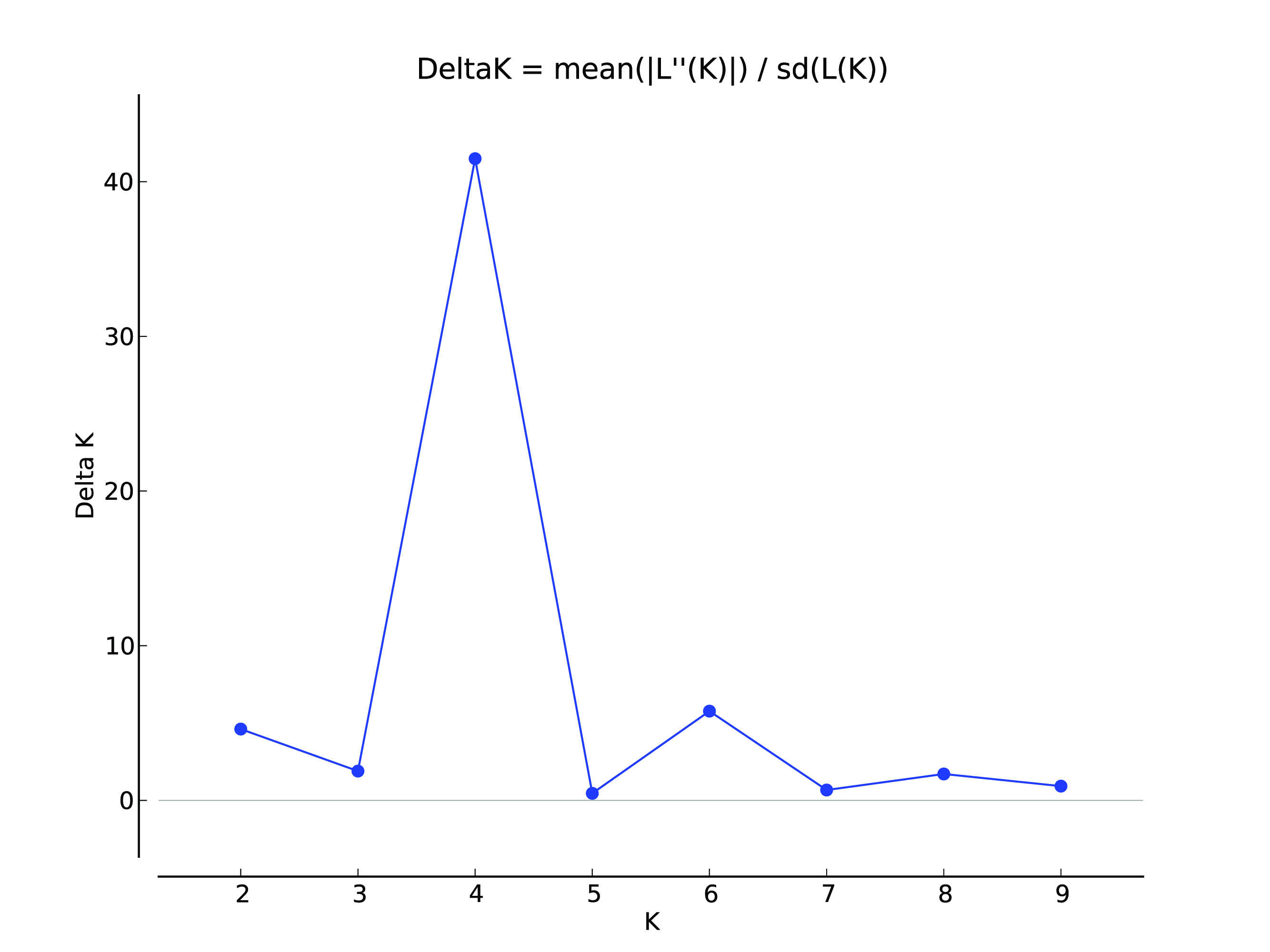

Supplement: Supplementary file 1 [file genes-13-01149-s001.zip › genes-1726515-supplementary/Supplementary Figure S2.tif]
